# ﻿Complete mitochondrial genome of *Rhodeuscyanorostris* (Teleostei, Cyprinidae): characterization and phylogenetic analysis

**DOI:** 10.3897/zookeys.1081.77043

**Published:** 2022-01-13

**Authors:** Wenjing Li, Ning Qiu, Hejun Du

**Affiliations:** 1 YANGTZE Eco-Environment Engineering Research Center, China Three Gorges Corporation, Beijing 100038, China YANGTZE Eco-Environment Engineering Research Center Beijing China; 2 Key Laboratory of Environmental Protection Technology on Water Transport, Ministry of Transport, Tianjin research institute for water transport engineering, M.O.T., Tianjin 300456, China Ministry of Transport, Tianjin research institute for water transport engineering Tianjin China; 3 Chinese Sturgeon Research Institute, China Three Gorges Corporation, Yichang, Hubei 443100, China Chinese Sturgeon Research Institute Yichang China

**Keywords:** Acheilognathinae, freshwater fish, genome structure, phylogenetic relationships

## Abstract

*Rhodeuscyanorostris* Li, Liao & Arai, 2020 is a freshwater fish that is endemic to China and restricted to Chengdu City in Sichuan Province. This study is the first to sequence and characterize the complete mitochondrial genome of *R.cyanorostris*. The mitogenome of *R.cyanorostris* is 16580 bp in length, including 13 protein-coding genes, two rRNA genes, 22 tRNA genes, and a control region (D-loop). The base composition of the sequence is 28.5% A, 27.6% C, 26.4% T, and 17.5% G, with a bias toward A+T. The genome structure, nucleotide composition, and codon usage of the mitogenome of *R.cyanorostris* are consistent with those of other species of *Rhodeus*. To verify the molecular phylogeny of the genus *Rhodeus*, we provide new insights to better understand the taxonomic status of *R.cyanorostris*. The phylogenetic trees present four major clades based on 19 mitogenomic sequences from 16 *Rhodeus* species. *Rhodeuscyanorostris* exhibits the closest phylogenetic relationship with *R.pseudosericeus*, *R.amarus*, and *R.sericeus*. This study discloses the complete mitochondrial genome sequence of *R.cyanorostris* for the first time and provides the most comprehensive phylogenetic reconstruction of the genus *Rhodeus* based on whole mitochondrial genome sequences. The information obtained in this study will provide new insights for conservation, phylogenetic analysis, and evolutionary biology research.

## ﻿Introduction

The cyprinid subfamily Acheilognathinae are small freshwater fish commonly known as bitterlings. These fish are characterized by their compressed body and their unique spawning strategy of depositing their eggs through extended spawning tubes into the gill cavity of live freshwater mussels and clams, where they hatch and develop until the juvenile fish are able to swim freely (Smith et al. 2004; Nelson et al. 2016; Li et al. 2017, 2020a). The subfamily Acheilognathinae includes 75 species and six valid genera, including *Acheilognathus*, *Paratanakia*, *Pseudorhodeus*, *Rhodeus*, *Sinorhodeus*, and *Tanakia* (Arai and Akai 1988; Chang et al. 2014; Li et al. 2017, 2020a). Most of the bitterlings inhabit still-water areas such as rivers, lakes, ponds, and reservoirs, and a few species live in streams. Bitterlings are omnivorous, mainly feeding on algae, plankton, and debris. All species are distributed in East and Southeast Asian countries (China, Korea, Japan, Vietnam, and Myanmar), except the three *Rhodeus* species in Europe and adjacent West Asia (Arai et al. 2001; Bogutskaya and Komlev 2001; Bohlen et al. 2006; Li et al. 2017; Bartáková et al. 2019).

Although the classification of the subfamily Acheilognathinae has been controversial for many years, the genus *Rhodeus* is distinguished from other genera by characteristics such as an incomplete lateral line, no barbels, two rows of light spots on the dorsal fin, a pharyngeal teeth formula of 0,0,5–5,0,0, a black spot on the anterior part of the dorsal fin in juveniles (absent in *R.amarus*, *R.meridionalis*, and *R.sericeus*), and wing-like yolk sac projections in the larvae (Arai and Akai 1988; Li et al. 2017; Li et al. 2020a; Li et al. 2020b). The genus *Rhodeus* is distributed in two disjunct regions of Eurasia and contains approximately 22 species/subspecies, with 19 in East Asia and three in Europe and West Asia (Arai et al. 2001; Bogutskaya and Komlev 2001; Bohlen et al. 2006; Li and Arai 2014; Bartáková et al. 2019; Li et al. 2020a). Among the 19 *Rhodeus* species/subspecies in East Asia, eight (*R.albomarginatus*, *R.cyanorostris*, *R.fangi*, *R.nigrodorsalis*, *R.ocellatus*, *R.sinensis*, *R.shitaiensis*, and *R.flaviventris*) have been reported in the Yangtze river basin of China (Li et al. 2020a, b).

*Rhodeuscyanorostris* Li, Liao & Arai, 2020 is endemic to China and is restricted to Chengdu City, Sichuan Province. It can be easily distinguished from other congeners (except for *R.nigrodorsalis*) by its blue snout, less branched dorsal- and anal-fin rays (both no more than eight of each), and lack of pored scales (Li et al. 2020a). Moreover, according to the personal observations of the first author, *R.cyanorostris* and *R.nigrodorsalis* are the only two bitterling species known to spawn mainly in winter, from January to March (Li et al. 2020a).

The mitochondrial genome has been widely used in molecular evolution, phylogeny, and population genetics because of its maternal inheritance, stable genetic composition, fast evolutionary rate, low recombination frequency, and highly conserved gene content (Ballard and Whitlock 2004; Oliveria et al. 2008; Galtier et al. 2009; Simon and Hadrys 2013; Hao et al. 2021; Zhao et al. 2021). Complete mitochondrial genomes can provide much more reliable phylogenetic information than smaller parts of the mitochondrial DNA (Huang et al. 2017; Hou et al. 2020) and have been considered reliable markers for constructing fish phylogenies in recent studies of the taxonomy and phylogeny of cyprinids (Wang et al. 2008; Tang et al. 2010; Muniyangdi et al. 2015; Huang et al. 2017; Chung et al. 2020; Zhang et al. 2021).

The main purpose of the current study is to disclose the complete mitochondrial genome sequence of *R.cyanorostris* for the first time and to construct a phylogenetic tree based on complete mitogenome sequences to elucidate the molecular phylogenetic relationship between *R.cyanorostris* and other species of *Rhodeus*. Therefore, this study provides essential scientific data and contributes to studies of the population genetics, adaptation, and phylogeny of *R.cyanorostris*.

## ﻿Materials and methods

### ﻿Sampling, sequencing, and assembly

Samples of *Rhodeuscyanorostris* were collected from the Pidu District of Chengdu City in the Sichuan Province of China (30°55'12"N, 103°50'51"E). The fish were caught with seines, anesthetized with MS-222 (Sigma, St. Louis, MO), fixed and stored in 95% ethanol. Species-level morphological identification was carried out according to the description of Fan Li (2020a). Total genomic DNA was extracted using a TIANamp Micro DNA Kit (Tiangen Biotech, Beijing, China) according to the manufacturer’s instructions. Then, DNA was stored at –20 °C for subsequent use.

The primers were designed based on the known mitochondrial genomes of *R.sinensis* by NCBI primer-BLAST (http://www.ncbi.nlm.nih.gov/tools/primer-blast/). PCR was performed by using an Eppendorf Thermal Cycler (5331AH760577, Eppendorf, Germany) with a 30 µL reaction mixture containing 15 µL of 2×Power Taq PCR MasterMix (Tianyi Huiyuan, China), 1 µL of DNA template, 1 µL of each primer (10 mM of each), and 12 µL of ultrapure water. The cycling procedures were as follows: denaturation at 95 °C for 5 min, 35 cycles of denaturation at 95 °C for 30 sec, annealing at 60 °C for 30 sec, extension at 72 °C for 1 min, and a final extension at 72 °C for 5 min. Agarose gel electrophoresis was used to detect each PCR product to verify the amplification efficiency. PCR products were purified and sequenced by primer walking from both directions.

Sequences were assembled using the DNASTAR package (Burland 2000). Overlapping fragments obtained by sequencing were edited using BIOEDIT v. 7.0.9.0 (Hall 1999) and aligned using MEGA v. 7.0 (Kumar et al. 2016).

### ﻿Mitogenome annotation and analyses

The mitogenome annotation, tRNA gene localization, and their secondary structure prediction of *R.cyanorostris* were all completed by the MITOS web server (http://mitos2.bioinf.uni-leipzig.de/index.py) (Bernt et al. 2013). The online MitoFish tool (http://mitofish.aori.u-tokyo.ac.jp/) was used to map the mitochondrial genome structure. The base structure, nucleotide composition, and relative synonymous codon usage (RSCU) were calculated using MEGA v. 7.0 (Kumar et al. 2016). The skewing of the nucleotide composition was calculated with the formulas: AT skew = (A – T) / (A + T) and GC skew = (G – C) / (G + C) (Perna and Kocher 1995). The complete mitochondrial genome sequence of *R.cyanorostris* has been submitted to NCBI (GenBank no. OL856007).

### ﻿Phylogenetic analyses

Twenty-one mitogenomic sequences downloaded from GenBank (Table 1) were aligned using MEGA v. 7.0 (alignment with CLUSTALW) with default settings (Kumar et al. 2016). The best model GTR +G + I was chosen based on the Akaike information criterion (AIC) using JMODELTEST v. 2 (Darriba et al. 2012), and the ML (maximum likelihood method) tree was constructed using PHYML v. 3.0 (Guindon et al. 2010). The confidence intervals were assessed through the bootstrap test inferred from 1000 replicates. An NJ (neighbor-joining method) tree was constructed based on the Kimura 2-parameter model with 1000 bootstrap replicates using MEGA v. 7.0 (Kumar et al. 2016).

**Table 1.** List of the species used to construct the phylogenetic tree.

Classific-ation	Subfamily	Genus	Species	Accession number	Gene length
Outgroup	Culterinae	*Hemiculter*	*Hemiculterleucisculus*	KF956522.1	16622 bp
Outgroup	Barbinae	*Onychostoma*	*Onychostomalepturum*	MT258556.1	16598 bp
Ingroup	Acheilognathinae	*Rhodeus*	*Rhodeusalbomarginatus*	MW896838.1	16764 bp
Ingroup	Acheilognathinae	*Rhodeus*	*Rhodeusamarus*	AP011209.1	16607 bp
Ingroup	Acheilognathinae	*Rhodeus*	*Rhodeusatremius*	AP010778.1	17282 bp
Ingroup	Acheilognathinae	*Rhodeus*	*Rhodeusatremiusatremius*	AP011255.1	16734 bp
Ingroup	Acheilognathinae	*Rhodeus*	*Rhodeusfangi*	KF980890.1	16733 bp
Ingroup	Acheilognathinae	*Rhodeus*	*Rhodeuslighti*	KM232987.1	16677 bp
Ingroup	Acheilognathinae	*Rhodeus*	*Rhodeusnotatus*	KU291171.1	16735 bp
Ingroup	Acheilognathinae	*Rhodeus*	*Rhodeusocellatuskurumeus*	AB070205.1	16674 bp
Ingroup	Acheilognathinae	*Rhodeus*	*Rhodeusocellatus* 1	DQ026430.1	16680 bp
Ingroup	Acheilognathinae	*Rhodeus*	*Rhodeusocellatus (Kner)* 2	KT004415.1	16761 bp
Ingroup	Acheilognathinae	*Rhodeus*	*Rhodeusocellatus* 3	MW007386.1	16675 bp
Ingroup	Acheilognathinae	*Rhodeus*	*Rhodeuspseudosericeus*	KF425517.1	16574 bp
Ingroup	Acheilognathinae	*Rhodeus*	*Rhodeussericeus*	KM052222.1	16581 bp
Ingroup	Acheilognathinae	*Rhodeus*	*Rhodeusshitaiensis*	KF176560.1	16774 bp
Ingroup	Acheilognathinae	*Rhodeus*	*Rhodeussinensis*	KF533721.1	16677 bp
Ingroup	Acheilognathinae	*Rhodeus*	*Rhodeussuigensis*	EF483934.1	16733 bp
Ingroup	Acheilognathinae	*Rhodeus*	*Rhodeusuyekii* 1	DQ155662.1	16817 bp
Ingroup	Acheilognathinae	*Rhodeus*	*Rhodeusuyekii* 2	EF483937.1	16827 bp

## ﻿Results

### ﻿Mitochondrial genomic structure and composition

**Figure 1.**
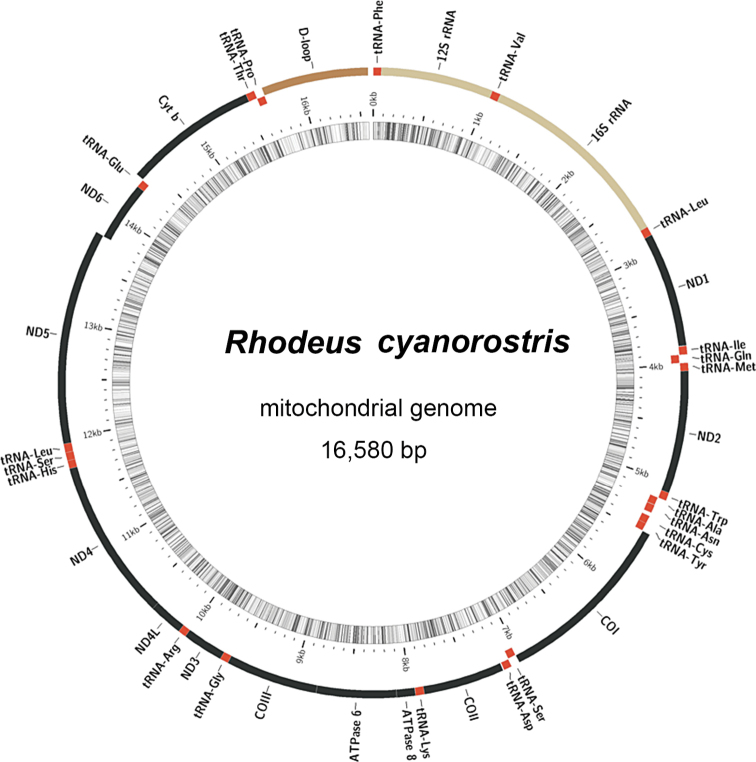
Gene map of the mitochondrial genome of *Rhodeuscyanorostris*. The genome contained two rRNA genes (in yellow), 13 coding genes (in black), 22 tRNA genes (in red), and a control region (D-loop) (in brown).

The complete mitochondrial genome of *Rhodeuscyanorostris* had a total length of 16580 bp (Fig. 1). The complete *R.cyanorostris* genome had a typical circular molecular structure and contained 37 genes, including 13 protein-coding genes (PCGs), two ribosomal RNA (rRNA) genes, 22 tRNA genes, and a noncoding control region (D-loop) (Table 2). Among these genes, NADH dehydrogenase 6 (ND6) and 8 tRNA genes (tRNA^Gln^, tRNA^Ala^, tRNA^Asn^, tRNA^Cys^, tRNA^Tyr^, tRNA^Ser^, tRNA^Glu^, tRNA^Pr^°) were encoded by L-strand, and the rest were encoded by H-strand. The mitogenome was compact, with eight gene overlaps, ranging in length from 1 to 7 bp. In addition, there were fourteen 1–30 bp coding gene spacer regions, with a total length of 63 bp; the longest spacer region fell between tRNA^Val^ and 16S rRNA genes.

**Table 2.** Organization of the mitochondrial genome of *Rhodeuscyanorostris*.

Locus	position		Size (bp)	Intergenic nucleotides	Codon		Anti-codon	Strand
	start	stop			start	stop		
tRNA^Phe^	1	69	69	0	–	–	GAA	H
12s rRNA	70	1026	957	1	–	–	–	H
tRN^AVa^l	1028	1099	72	30	–	–	TAC	H
16s rRNA	1130	2786	1657	0	–	–	–	H
tRNA^Leu^	2787	2862	76	0	–	–	TAA	H
*ND1*	2863	3837	975	4	GTG	TAA	–	H
tRNA^lle^	3842	3913	72	–2	–	–	GAT	H
tRNA^Gln^	3912	3982	71	1	–	–	TTG	L
tRNA^Met^	3984	4052	69	0	–	–	CAT	H
*ND2*	4053	5099	1047	–2	ATG	TAG	–	H
tRNA^Trp^	5098	5168	71	1	–	–	TCA	H
tRNA^Ala^	5170	5238	69	1	–	–	TGC	L
tRNA^Asn^	5240	5312	73	2	–	–	GTT	L
tRNA^Cys^	5345	5413	69	0	–	–	GCA	L
tRNA^Tyr^	5414	5483	70	1	–	–	GTA	L
*COI*	5485	7035	1551	0	GTG	TAA	–	H
tRNA^Ser^	7036	7106	71	2	–	–	TGA	L
tRNA^Asp^	7109	7178	70	9	–	–	GTC	H
*COII*	7188	7878	691	0	ATG	T(AA)	–	H
tRNA^Lys^	7879	7953	75	1	–	–	TTT	H
*ATP8*	7955	8119	165	–7	ATG	TAG	–	H
*ATP6*	8113	8796	684	–1	ATG	TAA	–	H
*COIII*	8796	9580	785	–1	ATG	TA(A)	–	H
tRNA^Gly^	9580	9650	71	0	–	–	TCC	H
ND3	9651	9999	349	0	ATG	T(AA)	–	H
tRNA^Arg^	10000	10069	70	0	–	–	TCG	H
*ND4L*	10070	10366	297	–7	ATG	TAA	–	H
*ND4*	10360	11738	1379	3	ATG	TA(A)	–	H
tRNA^His^	11742	11810	69	0	–	–	GTG	H
tRNA^Ser^	11811	11879	69	1	–	–	GCT	H
tRNA^Leu^	11881	11953	73	0	–	–	TAG	H
*ND5*	11954	13789	1836	–4	ATG	TAG	–	H
*ND6*	13786	14307	522	0	ATG	TAA	–	L
tRNA^Glu^	14308	14376	69	6	–	–	TTC	L
Cyt *b*	14383	15523	1141	0	ATG	T(AA)	–	H
tRNA^Thr^	15524	15597	74	–1	–	–	TGT	H
tRNA^Pr^°	15597	15666	70	54	–	–	TGG	L
D-loop	15721	16438	718	142	–	–	–	H

The base composition of the entire sequence was in the order of A (28.5) > C (27.6) > T (26.4) > G (17.5), with a bias toward A+T. This bias was observed in all genetic elements except for ND3 (Table 3). The complete genome also showed a clear AC bias (AT skew = 0.04, GC skew = –0.22), indicating a greater abundance of A than T and C than G (Table 3).

**Table 3.** Nucleotide contents of genes and the mitochondrial genome skew of *Rhodeuscyanorostris*.

Regions	Size (bp)	T	C	A	G	A+T (%)	G+C (%)	AT skew	GC skew
rRNAs	2645	20.0	25.1	33.4	21.5	53.4	46.6	0.25	–0.08
ND1	975	27.3	29.7	26.2	16.8	53.5	46.5	–0.02	–0.28
tRNAs	1562	26.6	22.0	28.6	22.9	55.2	44.9	0.04	0.02
ND2	1045	26.6	31.8	26.9	14.7	53.5	46.5	0.01	–0.37
COI	1551	29.3	27.3	24.3	19.0	53.6	46.3	–0.09	–0.18
COII	691	26.9	27.5	27.9	17.7	54.8	45.2	0.02	–0.22
ATP8	165	27.3	26.7	33.3	12.7	60.6	39.4	0.10	–0.36
ATP6	683	29.6	30.5	25.6	14.3	55.2	44.8	–0.07	–0.36
COIII	784	29.7	27.0	24.1	19.1	53.8	46.1	–0.10	–0.17
ND3	349	28.1	31.2	20.6	20.1	48.7	51.3	–0.15	–0.22
ND4L	297	28.6	30.0	24.6	16.8	53.2	46.8	–0.08	–0.28
ND4	1382	27.6	28.8	27.3	16.3	54.9	45.1	–0.01	–0.28
ND5	1836	27.9	28.2	29.6	14.2	57.5	42.4	0.03	–0.33
ND6	522	37.7	12.6	14.9	34.7	52.6	47.3	–0.43	0.47
Cyt *b*	1141	29.4	29.3	25.1	16.3	54.5	45.6	–0.08	–0.29
D-loop	860	31.6	21.9	30.9	15.6	62.5	37.5	–0.01	–0.17
PCGs	11421	28.7	28.1	25.9	17.3	54.6	45.4	–0.05	–0.24
Genome	16580	26.4	27.6	28.5	17.5	54.9	45.1	0.04	–0.22

Among the 13 protein-coding genes, the ND1 and COI genes started with GTG, while all other PCGs contained the usual ATG start codon. Eight of the 13 PCGs were terminated with the conventional stop codons (TAA or TAG), while the other five (ND4, COIII, COII, ND3, and Cyt *b*) were terminated with incomplete stop codons (TA or T). Moreover, the AT skew and GC skew values of the PCGs were –0.05 and –0.24, respectively, indicating that the nucleotides T and C had a greater abundance than their respective counterparts (Table 3).

**Figure 2.**
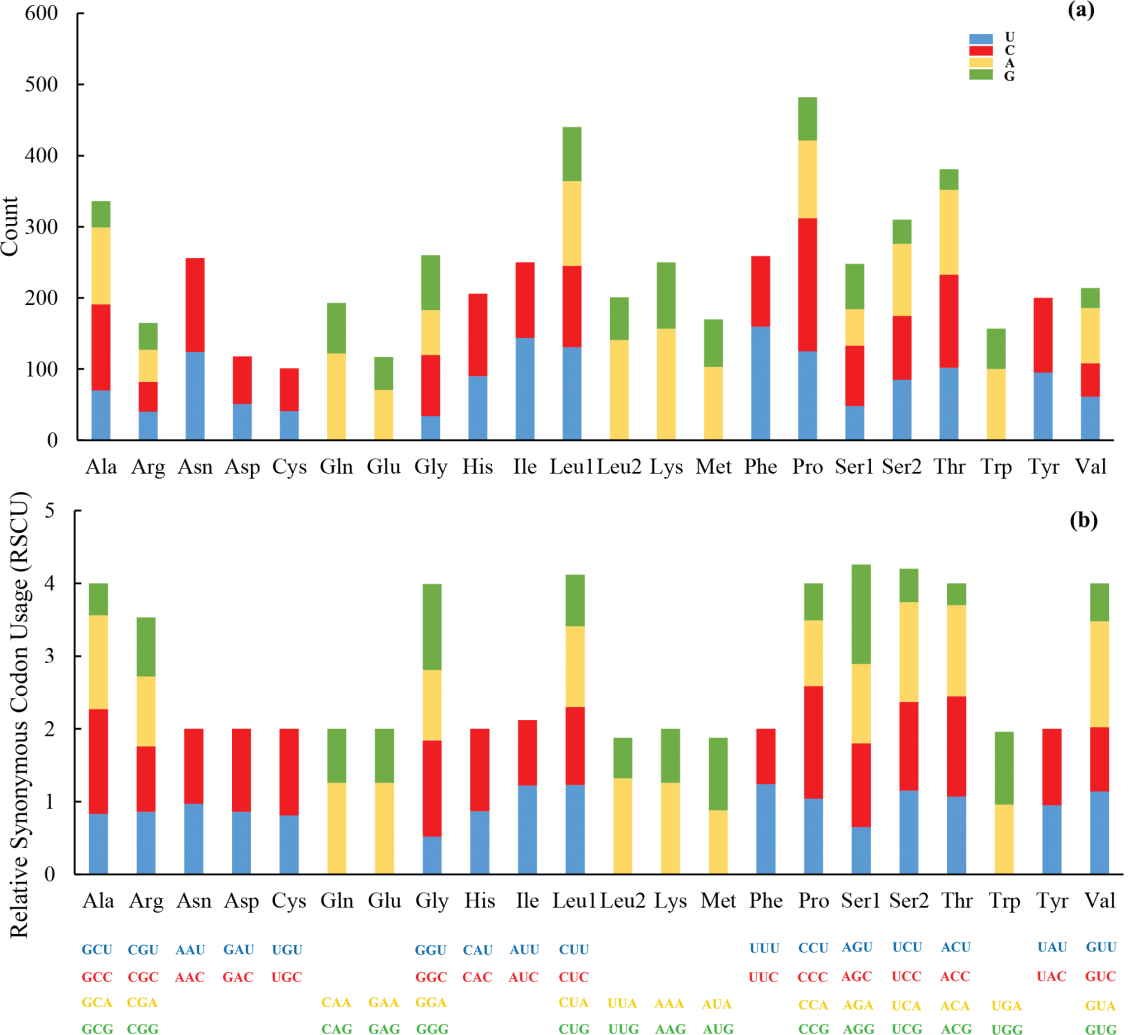
Codon distribution **a** and relative synonymous codon usage (RSCU) **b** in the mitogenome of *Rhodeuscyanorostris*.

Statistics on the relative synonymous codon usage (RSCU) of *R.cyanorostris* showed that the most abundant codons were CCC (Pro), UUU (Phe), AAA (Lys), and AUU (Ile) (Fig. 2).

### ﻿Transfer and ribosomal RNA genes

The two ribosomal RNAs (12S and 16S ribosomal RNA) were positioned between tRNA^phe^ and tRNA^leu^ and separated by tRNA^val^ in the mitogenome of *R.cyanorostris*. The 12S ribosomal RNA was composed of 957 bp, and the 16S ribosomal RNA was 1657 bp long. Both rRNA genes were encoded on the H-strand and displayed a positive AT skew and a negative GC skew (AT skew = 0.25, GC skew = –0.08).

**Figure 3.**
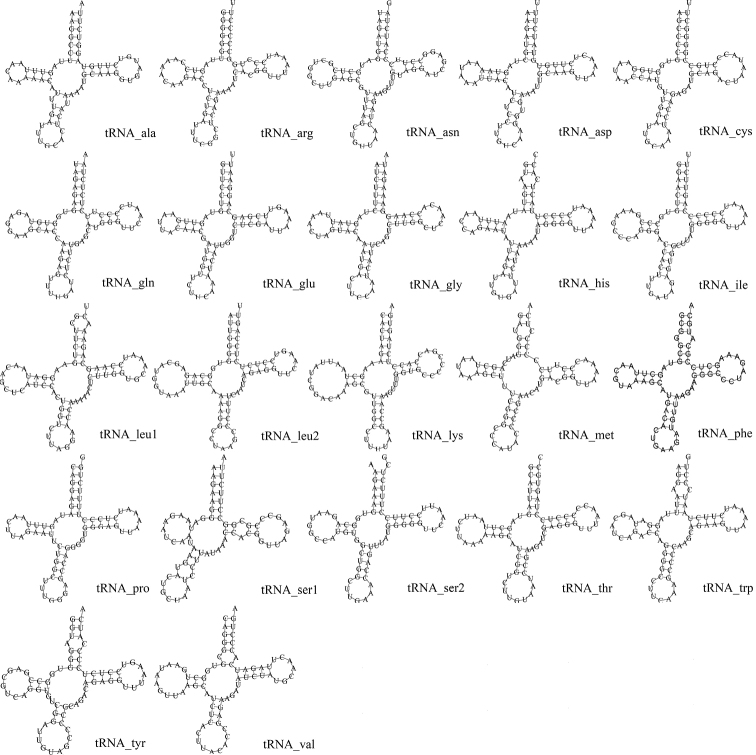
Putative secondary structures of the 22 tRNAs of *Rhodeuscyanorostris*.

The mitogenome of *R.cyanorostris* included 22 transfer RNA genes as in most vertebrates. These transfer RNA genes ranged from 69 to 76 bp. The total concatenated length of tRNA genes was 1562 bp, the AT skew of 22 tRNAs was 0.04, and the GC skew was 0.02, showing slightly higher A and G (Table 3). The secondary structures of all tRNA genes were traditional cloverleaf structures (Fig. 3). In addition to the typical base pairs (G-C and A-U), there were also some wobble G-U pairs in these secondary structures, which could form stable chemical bonds between U and G.

### ﻿Phylogenetic analysis

**Figure 4.**
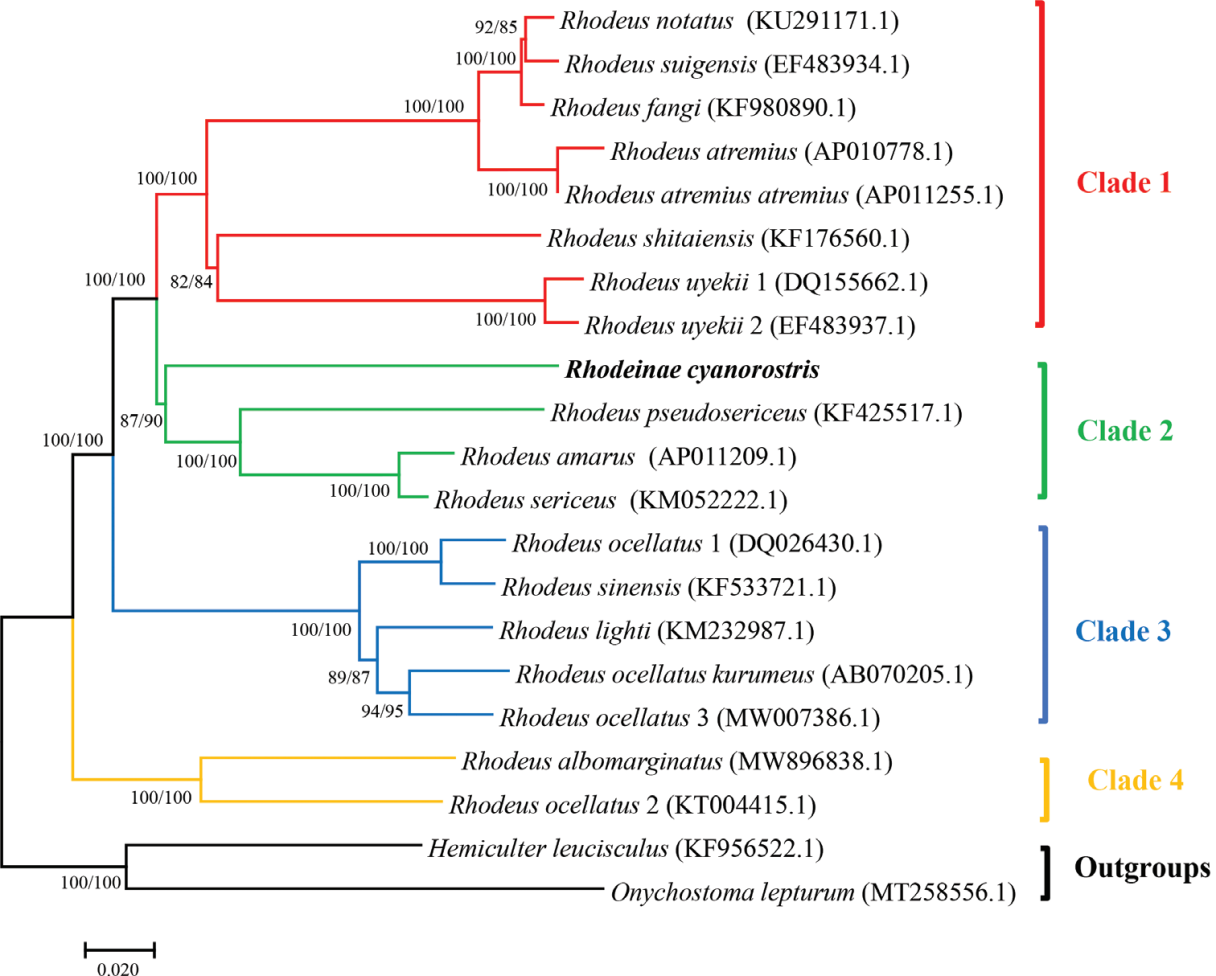
Phylogenetic trees derived from the maximum-likelihood (ML) and neighbor joining (NJ) approaches based on whole mitochondrial genomes. The numbers on the nodes are the bootstrap values of ML and NJ. The number after the species name is the GenBank accession number.

To elucidate the phylogenetic relationship in the genus *Rhodeus*, 21 whole mitochondrial genome sequences of 18 species were used in this study. As a result, ML and NJ analyses generated the same topological structure with well-supported values, and both presented four major sister clades (Fig. 4). Within Clade 1, the branch including three species (*R.notatus*, *R.suigensis*, and *R.fangi*) first formed a sister cluster with high bootstrap values with the branch containing *R.atremius* and *R.atremiusstremius*. Then, they clustered with the branch including *R.shitaiensis* and *R.uyekii*. In Clade 2, *R.cyanorostris* clustered together with *R.pseudosericeus*, *R.amarus*, and *R.sericeus*. In Clade 3, the branch including two species (*R.ocellatus* and *R.sinensis*) first formed a sister cluster with the branch containing *R.lighti*, *R.ocellatuskurumeus*, and *R.ocellatus* 3. Clade 4 included *R.albomarginatus* and *R.ocellatus* 2. *R.cyanorostris* exhibited the closest phylogenetic relationship with *R.pseudosericeus*, *R.amuarus*, and *R.sericeus*.

## ﻿Discussion

We successfully sequenced and assembled for the first time the mitogenome of *Rhodeuscyanorostris*, an endemic fish species in China. The mitogenome was 16580 bp in length, which was similar to the genome size of the known acheilognathine mitogenomes, for example, 16677 bp in *R.sinensis*, 16677 bp in *R.lighti*, and 16581 bp in *R.sericeus* (Wang et al. 2014; Xu et al. 2015; Yang et al. 2015). Differences in mitochondrial genome length in related species may be caused by changes in tandem repeats in the control region (Wang et al. 2020). Consistent with the genome structure of other teleost fish, the mitogenome of *R.cyanorostris* included 13 protein-coding genes (PCGs), 2 rRNA genes, 22 tRNA genes, and a non-coding control region (D-loop). The gene distribution was mainly presented on the H-strand, and only the ND6 gene and eight rRNA genes were located on the L-strand. This distribution is consistent with that of other species of Acheilognathinae (Wang et al. 2014; Xu et al. 2015; Yang et al. 2015). In comparison, the 13 PCGs in the mitogenome revealed a relatively low GC content, which was common in the *Rhodeus* mitogenome (Xu et al. 2015; Yang et al. 2015).

The whole mitochondrial genome of the genus *Rhodeus* is extremely similar in its nucleotide composition and codon usage, but there were also subtle differences. For example, among the 13 protein-coding genes of *R.cyanorostris*, two genes (ND1 and COI) start with GTG, and the other 11 start with ATG. In *R.shitaiensis*, only COI and ND5 start with GTG (Li et al. 2013). *Rhodeuslighti*, *R.sericeus*, *R.sinensis*, *R.suigensis*, and *R.uyekii* all start with ATG except for COI, which starts with GTG (Kim et al. 2006; Hwang et al. 2013; Wang et al. 2014; Xu et al. 2015; Yang et al. 2015). The termination codons of *R.lighti*, *R.sericeus*, *R.sinensis*, *R.suigensis*, and *R.uyekii* include conventional codons (TAA and TAG) and incomplete codons (T- and TA-).

The secondary structures of tRNA for *R.cyanorostris* are conserved, and these features meet the characteristics of vertebrate mitochondrial genomes (Zhao et al. 2021). In addition to the typical Watson-Crick pairing (A-U and G-C), there are also some typical pairings such as U-G. Some scholars have proposed that the non-Watson-Crick matched tRNAs can be transformed into fully functional proteins through a post-transcriptional mechanism (Pons et al. 2014; Zhao et al. 2021).

Mitochondrial genome sequences are widely used to study phylogenetic relationships because they offer small, stable changes over a long period for any given taxon. In this regard, whole mitochondrial genes can better transmit phylogenetic information than single genes (mitochondrial/nuclear) can (Huang et al. 2017; Hou et al. 2020). Previous studies have revealed different phylogenetic relationships of different bitterlings by using different molecular datasets. For the first time, we used whole mitochondrial genome sequences to construct the most comprehensive phylogenetic reconstruction of the genus *Rhodeus* thus far. The phylogenetic results indicated that there were some slightly different topologies compared to other studies due to different outgroups, contrast species, and molecular markers (Okazaki et al. 2001; Chang et al. 2014; Cheng et al. 2014; Kawamura et al. 2014). For example, Okazaki et al. (2001) reported the phylogenetic relationships of 27 species or subspecies of Acheilognathinae based on the 12S rRNA gene. Chang et al. (2014) used six nuclear gene loci (RAG1, RH, IRBP2, EGR1, EGR2B, and EGR3) and one mitochondrial gene (cyt *b*) to study the phylogenetic relationship of the subfamily Acheilognathinae, including *Rhodeus*. Cheng et al. (2014) reconstructed a species-level phylogenetic tree of Acheilognathinae based on the mtDNA cyt *b* and 12S rRNA gene sequences. Kawamura et al. (2014) elucidated the phylogeny of 49 species or subspecies in three genera (*Tanakia*, *Rhodeus*, and *Acheilognathus*) with cyt *b.* In this study, the phylogenetic tree showed that the genus *Rhodeus* is divided into four clades. *Rhodeuscyanorostris* is most closely related to *R.pseudosericeus*, *R.amarus*, and *R.sericeus*. They occupy Clade Ⅱ, and the closer phylogenetic relationship between the latter three was consistent with the study of Kawamura et al. (2014). The mitogenome sequences of *R.shitaiensis*, *R.uyekii*, and four members of the *R.smithii* complex (*R.fangi*, *R.notatus*, *R.atremius*, *R.suigensis*) (Kimura and Nagata 1992; Arai et al. 2001; Okazaki et al. 2010; Yu et al. 2016) constituted Clade Ⅰ of the phylogenetic tree. Furthermore, the phylogenetic relationship among species was also closely related to their morphological similarity. For example, *R.albomarginatus* is the most morphologically similar to *R.ocellatus* and *R.sinensis* (Chang et al. 2014), which are the most widely distributed *Rhodeus* species in China. They occupied the Clades Ⅲ and Ⅳ. According to Li et al. (2010), *R.shitaiensis* closely resembled the *R.sericeus* complex (*R.sericeus*, *R.colchicus*, *R.meridionalis*, and *R.amarus*).

## ﻿Conclusions

In summary, we successfully sequence and characterize the complete mitochondrial genome sequence of *Rhodeuscyanorostris* for the first time and furtherly elucidate the relationship between *R.cyanorostris* and other species in the genus *Rhodeus*. The information obtained from this study will be valuable in further studies on the conservation, molecular identification, and evolutionary biology of the diverse *Rhodeus* species.
